# Clinical and Molecular Profiles and Treatment Outcomes in Patients With Acute Promyelocytic Leukaemia: A Single-Centre Experience

**DOI:** 10.7759/cureus.92377

**Published:** 2025-09-15

**Authors:** Omkar K Choudhari, Priyanka Soni, Lalit M Sharma, Disha Satya, Satyajeet Soni, Naveen Gupta, Ajay Yadav, Hemant Malhotra, Purvish M Parikh

**Affiliations:** 1 Clinical Hematology, Mahatma Gandhi Medical College and Hospital, Jaipur, IND; 2 Medical Oncology, Mahatma Gandhi Medical College and Hospital, Jaipur, IND

**Keywords:** acute promyelocytic leukaemia (apml), all-trans retinoic acid (atra), arsenic trioxide, blood transfusions, differentiation syndrome

## Abstract

Introduction: Acute promyelocytic leukaemia (APL) is one of the most treatable blood cancers with a high cure rate. The reciprocal translocation t (15; 17) results in the formation of the promyelocytic leukaemia-retinoic acid receptor alpha (PML-RARα) gene and its fusion protein, which are hallmarks of this disease. Patients often present with bleeding diathesis and may experience a fulminant early course. Outcomes with all-trans retinoic acid-based regimens are very favourable. In this study, we share our experience with APL at our centre.

Objective: The study aimed to study clinical and molecular profiles and treatment outcomes in patients with APL.

Materials and methods: We collected data on all patients diagnosed with and treated for APL at our centre between June 2019 and December 2024. APL was diagnosed based on the PML-RARα mutation and identified by reverse transcriptase real-time polymerase chain reaction or fluorescent in situ hybridisation, along with bone marrow aspiration morphology and immunophenotyping by flow cytometry. Baseline data, including demographic, clinical and molecular characteristics, therapy details, complications, and outcomes, were recorded. Baseline risk stratification was performed using the modified Sanz criteria.

Results: A total of 40 patients with APL were evaluated. The male-to-female ratio was 3:1. Patients' ages ranged from 4 to 79 years, with a median age of 37 years. The most common presenting feature was fever (55%), followed by bleeding (35%), dyspnoea (15%), generalised weakness (7.5%), and altered sensorium (2.5%). According to the modified Sanz criteria, 23 (57.5%) patients were classified as low risk, while 17 (42.5%) were classified as high risk. The PML-RARα fusion gene was detected in all patients, with the distribution of breakpoint cluster region 1 (bcr1), bcr2, and bcr3 transcripts being 20 (50%), 1 (2.5%), and 19 (47.5%), respectively. Coagulation parameters did not significantly differ between low- and high-risk groups. Twenty-nine (72.5%) patients received all-trans retinoic acid (ATRA) and arsenic trioxide (ATO)-based induction chemotherapy, while 11 (27.5%) received ATRA, ATO, and daunorubicin-based induction chemotherapy. Differentiation syndrome occurred more frequently in the high-risk group than in the low-risk group (p=0.001). The most common complication during induction chemotherapy was febrile neutropenia (97.5%). The transfusion requirement during induction was higher for bcr1 and bcr3 transcripts, but this did not reach statistical significance (p>0.05). Morphological remission was achieved in 92.5% of patients. Induction mortality was 7.5%. All patients except one received ATRA and ATO-based consolidation. No mortality was observed during consolidation. At the end of consolidation, 92.5% of patients were in morphological and molecular remission. A total of 16 (40%) patients received maintenance treatment with ATRA, 6-mercaptopurine, and methotrexate. Relapse was observed in 2 (5%) patients. At a median follow-up of 30 months, the event-free survival of the entire cohort was 87.5%.

Conclusion: Most patients with APL presented with fever and bleeding. The bcr1 transcript of PML-RARα was the most commonly observed. ATRA and ATO-based treatment was associated with high remission rates, manageable toxicity, and a low relapse rate.

## Introduction

Acute promyelocytic leukaemia (APL) is one of the treatable blood cancers with a high cure rate. The reciprocal translocation, t (15; 17), leads to the formation of the promyelocytic leukaemia-retinoic acid receptor alpha (PML-RARα) gene and fusion protein, which is the hallmark of APL. The promyelocytic leukaemia (PML) gene acts as a tumour suppressor gene and plays a role in apoptosis, while the retinoic acid receptor alpha (RARα) gene is involved in myeloid differentiation. Retinoic acid receptors mediate differentiation in many tissues. RARα is expressed on hematopoietic stem cells, which induces differentiation of promyelocytes. The PML-RARα fusion blocks retinoic acid receptor-induced differentiation, leading to the accumulation of abnormal promyelocytes and the subsequent development of APL [1]. APL manifests as a hypergranular and microgranular variant morphologically. The hypergranular variant presents with a low total leucocyte count (TLC) and a high propensity for disseminated intravascular coagulation (DIC). Conversely, the microgranular variant presents with a high TLC and an increased risk of differentiation syndrome [2]. Patients with APL often present with bleeding diathesis and may experience a fulminant course in the early days of therapy with all-trans retinoic acid (ATRA)-induced differentiation syndrome. The induction therapy in APL is generally associated with a substantial requirement for blood products, along with monitoring of coagulation parameters [3]. Complications during induction therapy include various infections, differentiation syndrome, pleural and pericardial effusions, bleeding diathesis, febrile neutropenia (FN), and mortality [4]. With the advent of PML-RARα degradation-directed treatment using ATRA and arsenic trioxide (ATO), the function of PML-RARα can be modulated. There are three breakpoint cluster regions (bcr) in intron 6, exon 6, and intron 3 of the PML gene, as well as one in intron 2 of the RARα gene, along with the presence of some atypical isoforms [5]. Presently, there is a paucity of data on APL in the Indian population. Hence, this study is undertaken to understand the biology of disease, molecular profiling, and treatment outcomes in the Indian population.

## Materials and methods

We collected ambispective data from the medical records of all patients diagnosed with and treated for APL between June 2019 and December 2024 in the Department of Clinical Hematology, Mahatma Gandhi Medical College and Hospital, Jaipur, India, a tertiary care centre in northwestern India. APL was diagnosed based on the PML-RARα fusion, which was identified by reverse transcriptase real-time polymerase chain reaction (RT-PCR) or fluorescent in situ hybridisation (FISH), with the presence of t (15; 17), along with bone marrow aspiration morphology and immunophenotyping by flow cytometry. Baseline data, including demographic profiles, clinical characteristics at initial presentation, and additional factors such as details of therapy, complications, and outcomes, were recorded and analysed. Patients with a TLC >10,000/µL were classified as high-risk APL, while those with a TLC <10,000/µL were classified as low-risk APL, according to the modified Sanz Criteria [6]. All patients received ATRA and ATO-based induction. Chemotherapy was given as per the clinicians’ discretion. Post-induction treatment consisted of ATRA with either ATO or chemotherapy. All high-risk patients received maintenance therapy.

Low-risk APL patients received ATRA 45 mg/m²/day (25 mg/m² for children) in two divided doses from day 1 to 45, with ATO 0.15 mg/kg from day 9 to 45. High-risk patients additionally received daunorubicin 60 mg/m² on days 1 to 3. Consolidation involved ATRA (45 mg/m²/day; 25 mg/m² for children) administered on days 1-14 and 29-42, plus ATO (0.15 mg/kg) on days 1-5, 8-13, 15-20, and 22-27 in a 56-day cycle, repeated for four cycles. Maintenance consisted of ATRA (45 mg/m²/day for 14 days), followed by 6-mercaptopurine (50 mg/m²/day) and methotrexate (15 mg/m² weekly), for 10 weeks and continued for 2 years.

Patients were closely monitored for differentiation syndrome, pseudotumour cerebri, electrocardiography (ECG) abnormalities, and electrolyte levels (calcium, magnesium, and potassium). The target platelet count was ≥50 × 10⁹/L and fibrinogen ≥150 mg/dL, maintained through cryoprecipitate or fresh frozen plasma (FFP) transfusions. ECGs were performed weekly; ATO was withheld if QTc exceeded 500 ms. Prophylactic antibiotics, antifungals, and antivirals were administered according to institutional protocols. Differentiation syndrome, characterised by fever, weight gain, respiratory distress, and pulmonary infiltrates, was treated with IV dexamethasone 10 mg twice daily until resolution, then followed by oral prednisolone (0.5-1 mg/kg). ATRA was withheld or dose-adjusted and resumed after improvement. High-risk patients received prednisolone at 0.5 mg/kg/day as prophylaxis against differentiation syndrome.

Data from APL patients were collected up to the last follow-up. Complete remission was defined as the absence of clinical evidence of APL, normalisation of peripheral blood counts (platelets >100 × 10^9^/L and absolute neutrophil count >1 × 10^9^/L), and a bone marrow with less than 5% blasts plus promyelocytes. Molecular remission was defined as a negative PML-RARα result on RT-PCR. Overall survival was calculated from the date of diagnosis to the last follow-up or the date of death. Molecular relapse was defined as the reappearance of PML-RARα transcripts on RT-PCR (sensitivity 10⁻⁴). RT-PCR was performed every 3 months for 2 years, then every 6 months afterwards.

Statistical analysis was conducted using Python 3 (Python Software Foundation, Wilmington, DE, USA). Descriptive statistics were summarised with means and standard deviations or medians and interquartile range (IQR), depending on the normality of the distribution. Variables were compared between low- and high-risk groups, as well as among transcripts bcr1, bcr2, and bcr3, using Welch's t-test, the Mann-Whitney U test, or the Fisher exact test, as appropriate. A p-value <0.05 was considered statistically significant. Survival analysis used Kaplan-Meier curves, and the log-rank test was employed to compare overall survival rates. Overall survival was defined as the time from presentation and diagnosis until death from any cause. Event-free survival was defined as the time from presentation to any event, relapse, or death.

## Results

A total of 40 patients with APL were evaluated. Thirty (75%) were male and ten (25%) were female. Patients' ages ranged from 4 to 79 years, with a median age of 37 years (IQR: 30.25-49.75 years). The most common presenting feature was fever (55%), followed by bleeding (35%), dyspnoea (15%), generalised weakness (7.5%), and altered sensorium (2.5%). The most frequent sites of bleeding included gum bleeding (22.5%), bleeding per vaginam (7.5%), haemoptysis (5%), rectal bleeding (5%), and haematuria (2.5%). The duration of symptoms varied from 5 to 15 days, with a median time to presentation of 7 days (IQR: 5-10 days). Eight (20%) patients had a history of previous transfusion before presenting to the hospital. Haemogram revealed that 39 (97.5%) patients were anaemic (haemoglobin: <12 g/dL for women, <13 g/dL for men, and <10.9 g/dL for 4-year-olds). The presenting platelet count in the participants ranged from 8,000/μL to 88,000/μL. Based on the modified Sanz criteria, 23 (57.5%) patients were classified as low risk, while 17 (42.5%) were classified as high risk. General characteristics of patients in the low- and high-risk groups are detailed in Table 1.

**Table 1:** Patients’ general characteristics with presenting complaints *p-value <0.05. To compare means, the Welch t-test was used, while to compare medians, the Mann-Whitney U test was used. APTT, Activated partial thromboplastin time; PT, Prothrombin time; TLC, Total leucocyte count

Parameters	Low risk, N (%)	High risk, N (%)	p-value
Gender			
Male	19 (82.60%)	11 (64.70%)	-
Female	4 (17.39%)	6 (35.29%)	-
Number of patients	23	17	
Presenting complaints			
Fever	13 (56.52%)	10 (58.82%)	-
Breathlessness	2 (8.69%)	4 (23.52%)	-
Bleeding			
Haemoptysis	4 (17.39%)	1 (5.88%)	-
Gum bleeding	3 (13.04%)	1 (5.88%)	-
Epistaxis	2 (8.69%)	1 (5.88%)	-
Wet bleeds	1 (4.34%)	3 (17.64%)	-
Bleeding PV	0	2 (11.76%)	-
Bleeding PR	1 (4.34%)	1 (5.88%)	-
Haematuria	1 (4.34%)	0	-
Laboratory parameters at diagnosis			
Haemoglobin (gm/dL)	8.8±2.35	7.88±1.48	0.14
TLC/µL	3191±1680	39290±37163	0.001*
Platelets/µL	49043±22542	27411±21139	0.24
PT (median)	14.2	16.3	0.75
APTT (median)	29.17	29.10	0.87
Fibrinogen (median)	235	157	0.76

The PML-RARα fusion gene was detected in all cases. The bcr transcripts observed in patients were bcr1 (50%), bcr2 (2.5%), and bcr3 (47.50%). Twenty-nine (72.5%) patients received ATRA and ATO-based induction, while 11 (27.5%) patients received ATRA, ATO, and daunorubicin-based induction chemotherapy. Differentiation syndrome was observed in 22 (55%) patients, with 2 (5%) patients succumbing to the complication. Two (5%) patients, one from each risk group, developed a thrombotic event: one experienced thrombosis in the left mid-brachial vein (arm without a peripherally inserted central catheter or PICC), and another had pulmonary embolism with left lower limb deep vein thrombosis (DVT). FN occurred in 97.5% of patients, with a median duration of 7 days (range: 5-15 days). Seventy-five per cent (30 patients) had grade III FN, while nine patients had grade II FN, according to the Common Terminology Criteria for Adverse Events (CTCAE). Other complications included pneumonia (40%), oral mucositis (20%), anal fissure (15%), retinal haemorrhage (10%), diarrhoea (5%), liver abscess (5%), sub-conjunctival haemorrhage (5%), bilateral lower limb cellulitis (2.5%), haemorrhoids (2.5%), bilateral bronchiolitis (2.5%), seborrhoeic dermatitis (2.5%), PICC line infection (2.5%), and thalamic bleed (2.5%). The QT interval was monitored on an ECG in all patients receiving ATRA and ATO. A total of 6 patients experienced QT prolongation along with transaminitis during treatment, necessitating dose interruption for a mean of 3 days, and no occurrence of QT prolongation was observed upon resuming the drug. Induction mortality was observed in 3 (7.5%) patients. Differentiation syndrome occurred more frequently in the high-risk group (82.35%) than in the low-risk group (34%) (p=0.008), as shown in Table 2.

**Table 2:** Treatment and complications in both study groups *p-value <0.05. To compare between the groups, Fisher's exact test was used. ATRA, All-trans retinoic acid; ATO, Arsenic trioxide; B/L, Bilateral; PICC, Peripherally inserted central catheter

Parameters	Low risk, N (%)	High risk, N (%)	p-value
Treatment received			
ATRA + ATO	20 (86.96%)	7 (41.18%)	-
ATRA + ATO + daunorubicin	3 (13.04%)	10 (58.82%)	-
Complications during induction chemotherapy			
Differentiation syndrome	15 (65%)	14 (82%)	0.001*
Pneumonia	11 (47.83%)	8 (47.06%)	1
Anal fissure	4 (17.39%)	2 (11.76%)	0.66
Retinal haemorrhage	4 (17.39%)	2 (11.76%)	0.66
Liver abscess	1 (4.35%)	1 (5.88%)	1
PICC line infection	0	1 (5.88%)	-
Sinusitis	1 (4.35%)	1 (5.88%)	1
Folliculitis	0	1 (5.88%)	-
Cellulitis B/L lower limb	0	1 (5.88%)	-
Herpes labialis	0	1 (5.88%)	-
Acute kidney injury	0	1 (5.88%)	-
Deep venous thrombosis	1 (4.35%)	00	-

**Figure 1:**
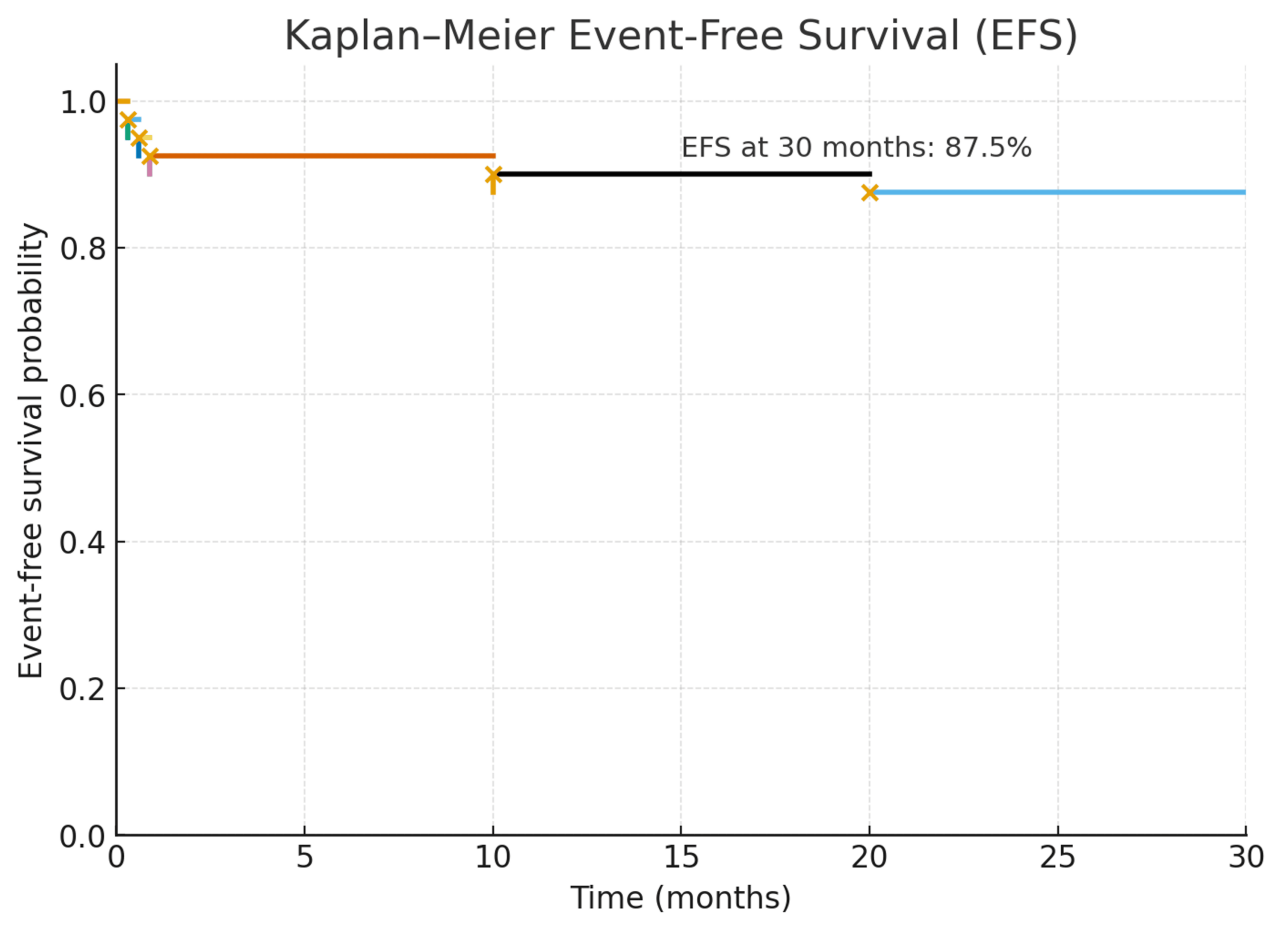
Event-free survival of patients in our study group

Transfusions of single-donor platelets (SDPs), packed red blood cells (PRBCs), cryoprecipitate, and FFP did not show significant differences between the bcr1 and bcr3 groups. When comparing isoforms of PML-RARα, patients with the bcr3 (5%) transcript exhibited higher mortality than those with the bcr1 (2.5%) transcript, although this difference is not statistically significant (p>0.05). Molecular remissions were maintained after consolidation, and induction mortality was low (3 patients). A total of 2 (5%) patients experienced relapse. The event-free survival rates at a median of 30 months were 87.5% for bcr3 and 95% for bcr1 (p=0.08), as shown in Figure 1. The estimated overall survival rate was 92.5% at the end of induction and consolidation. There was no significant difference in overall survival between low-risk and high-risk groups (p=0.38) nor between bcr1 and bcr3 (p=0.49).

Morphological remission was observed in 92.5% of patients, and molecular remission was achieved in 90.0% at the end of induction and in 92.5% at the end of consolidation. All patients received ATRA- and ATO-based consolidations. During consolidation, one patient experienced pancytopenia, which recovered spontaneously after ATO was withheld. Upon resuming, cytopenia did not recur. By the end of consolidation, all patients achieved molecular remission. High-risk patients (40%) underwent two years of maintenance therapy with ATRA, 6-mercaptopurine, and methotrexate. One high-risk patient experienced combined central nervous system (CNS) and medullary relapse during maintenance chemotherapy. He received fresh induction with ATRA, ATO, and triple intrathecal-based therapy, attained molecular remission, and underwent an autologous stem cell transplant. At the end of maintenance, all high-risk patients remained in morphological and molecular remission. One low-risk patient (2.5%) experienced medullary relapse after one year of completing consolidation; he underwent reinduction with ATRA + ATO followed by autologous stem cell transplant after achieving molecular and morphological remission. With a median follow-up of 30 months, death was recorded in 7.5% of patients. Post-induction follow-up showed that the median survival time had not been reached.

## Discussion

APL is a subtype of acute myeloid leukaemia with a favourable prognosis. Although rare, it is treatable, and long-term remission can be achieved. Prompt diagnosis and treatment with ATRA eliminates the molecular block and promotes further differentiation. Suspecting APL and initiating treatment not only reduces the risk of additional bleeding episodes but also increases the likelihood of differentiation syndrome. We evaluated 40 patients with varying presentations who were diagnosed with APL through molecular diagnosis. The initial demographic data, along with haemoglobin and platelet levels, were similar between high- and low-risk groups in another study by Zheng et al. [7].

In our study, many patients from both groups showed some form of bleeding, more often in the high-risk group; however, their coagulation parameters did not differ significantly. These findings aligned with another study by Simon et al. [8]. The most common complication during induction chemotherapy was differentiation syndrome, followed by pneumonia; these findings were comparable with those in another study by Yedla et al. [4]. Differentiation syndrome was identified in 29 patients across both groups. The risk of developing differentiation depends on the type of transcript; patients with the bcr3 transcript face a higher risk. Since patients with the bcr3 transcripts also require significant transfusions, which can lead to weight gain, they also have an increased likelihood of developing differentiation syndrome. However, in our study, the patient with the bcr1 transcript had the highest transfusion requirement [9,10].

APL carries increased risks of both thrombosis and bleeding. The cause of thrombosis in patients with abnormal coagulation parameters is explained by the activation of the coagulation cascade, along with precipitating factors such as immobility and the use of indwelling catheters. Moreover, some studies have shown that ATRA itself may cause thrombosis through unknown mechanisms [11]. In several other studies, the presence of the plasminogen activator inhibitor-1 4G/4G genotype has been associated with an increased risk of thrombosis. Corticosteroids used in the treatment of differentiation syndrome can also lead to increased von Willebrand factor levels and a hypercoagulable state. One of our patients experienced pulmonary embolism with DVT, while another had brachial vein thrombosis. Similar findings have been reported in other studies as well [12-14].

APL patients commonly present with bleeding. In our study, one patient exhibited a thalamic bleed without initiation of ATRA. A few other studies suggest that initiating ATRA may be associated with increased bleeding, likely due to heightened fibrinolysis mediated by plasminogen activators. Typically, when treatment with ATRA is introduced, it induces differentiation. This reduces the procoagulant tendency, but initial exposure leads to an increase in plasminogen activators, resulting in unopposed fibrinolysis, consumption coagulopathy, and DIC. In APL phenotypes, hypergranular variants are associated with a higher risk of DIC and bleeding tendencies, whereas microgranular variants generally do not experience haemorrhagic complications [15,16].

One patient from each risk group experienced a relapse. Extramedullary relapses are uncommon in APL, with isolated CNS relapses and cutaneous forms being observed [17]. One patient from the high-risk group had both CNS and medullary relapses and carried the FMS-like tyrosine kinase 3-internal tandem duplication mutation. Meanwhile, another patient from the low-risk group experienced a medullary relapse. This patient was not tested for other mutations, but both patients had the bcr3 PML-RARα transcript. Patients with FLT3 mutations are more likely to relapse, as shown by various studies [18-20]; however, the association of the bcr3 transcript with relapse has also been linked to a higher need for transfusions, as observed in our study and supported by research carried out by Baba et al. [19]. Furthermore, high-risk patients tend to relapse more frequently.

In studies from India, end of induction haematological remission was observed in 79% of patients, while 21% died during induction therapy, based on a retrospective analysis of 111 patients. The same study had a median follow-up of 34 months, with event-free survival at 69.3% and overall survival at 74.7% [21]. In our study, 37 (92.5%) patients achieved complete haematological remission at the end of induction, and 92.5% achieved haematological remission after consolidation. Event-free survival was 87.5% and overall survival was 92.5% over a median follow-up of 30 months. There was no significant difference in overall survival between low-risk and high-risk APL patients. Furthermore, in our study, patients with bcr3 transcripts showed more relapses and higher transfusion requirements, although these differences were not statistically significant. Another study from India, involving 33 patients, reported a complete remission rate of 81.81% at the end of induction, with relapse occurring in four patients (14.81%) at a median of 63 months, while 2-year overall survival and event-free survival were 75.13%±8.51% and 62.15%±1.08%, respectively [22]. In our study, the complete remission rates were higher (92.5%), with overall event-free survival and overall survival rates being 87.5% and 92.5%, respectively, at a median follow-up of 30 months.

Few studies have shown variable transfusion rates based on PML-RARα transcripts. In our research, patients with bcr1 transcripts required the highest amounts of PRBC, SDP, FFP, and cryoprecipitate transfusions, followed by those with other transcripts. However, this difference was not statistically significant. The transfusion requirements were high in both the bcr1 and bcr3 groups. The reasons for the increased transfusion needs in bcr3 may include leucocytosis, DIC, and a subsequent rise in transfusion due to activation of the coagulation cascade or through inflammatory mediators released because of leucocytosis [23].

Limitations

Since this was an ambispective study, retrospective data were extracted from the medical records, which have inherent limitations. Due to the small sample size, the results cannot be generalised to the population. Late relapses may not be documented because of the short follow-up period.

## Conclusions

Patients with APL exhibited high rates of bleeding and infections. The bcr1 and bcr3 transcripts of PML-RARα were most common and exhibited no major differences in clinical outcomes. ATRA and ATO-based induction and consolidation demonstrated high efficacy, low induction mortality, and a manageable toxicity profile. Low- and high-risk patients exhibited similar clinical outcomes, except for a higher rate of differentiation syndrome in the high-risk group. Deep and sustained remissions were achieved with an ATRA and ATO-based regimen.
